# Current Understanding of the Genetics of Intervertebral Disc Degeneration

**DOI:** 10.3389/fvets.2020.00431

**Published:** 2020-07-24

**Authors:** Peter J. Dickinson, Danika L. Bannasch

**Affiliations:** ^1^Department of Surgical and Radiological Sciences, School of Veterinary Medicine, University of California, Davis, Davis, CA, United States; ^2^Department of Population Health and Reproduction, School of Veterinary Medicine, University of California, Davis, Davis, CA, United States

**Keywords:** chondrodystrophy, fibroblast growth factor 4, heritable, intervertebral disc degeneration, retrogene

## Abstract

Premature degeneration of the intervertebral disc and its association with specific chondrodystrophic dog breeds has been recognized for over a century. Several lines of evidence including disease breed predisposition, studies suggesting heritability of premature intervertebral disc degeneration (IVDD) and association of a dog chromosome 12 (CFA 12) locus with intervertebral disc calcification have strongly supported a genetic component in IVDD in dogs. Recent studies documenting association of IVDD with an overexpressing *FGF4* retrogene on CFA 12 have opened up new areas of investigation to further define the pathophysiology of premature IVDD. While preliminary data from studies investigating FGF4 retrogenes in IVDD implicate FGF4 overexpression as a major disease factor, they have also highlighted knowledge gaps in our understanding of intervertebral disc herniation which is a complex and multifactorial disease process.

## Introduction

The list of inherited neurological disorders in companion and production animals is ever expanding. There are over 120 known genetic variants for neurological disorders in dogs alone (1), and with advances in molecular genetic technology and consistently decreasing costs, the list is continuing to expand at a rapid rate. Many of these disorders are associated with breed specific syndromes and have a relatively “localized” effect on the health of the overall dog population. The vast phenotypic diversity within domesticated dogs is the result of selection for genetic variants that define key traits such as skeletal size, body size, skull shape, snout length, coat color, leg length, and other breed-defining characteristics (2, 3). Beyond the “desirable” morphological traits, undesirable “disease” syndromes may be associated with these genetic loci due to either multiple phenotypic sequelae of specific variants, or associated genetic variants carried within long regions of linkage disequilibrium. This can be particularly problematic when disease causing genes define key characteristics of the breed e.g., leg length or head shape resulting in the variant being essentially fixed (homozygous for the associated allele) in a majority if not all animals within certain breeds; premature degeneration of the intervertebral disc (IVD) in chondrodystrophic dog breeds provides a quintessential example of this dilemma. The high penetrance of intervertebral disc disease (IVDD) associated genes in many popular dog breeds presents a daunting clinical challenge and results in millions, if not billions of dollars of annual veterinary treatment-related expense and suffering. However, as with the profound impact of preventative and screening practices in cancer medicine, the potential for genetic interventions to have dramatic effects on clinical IVDD in dogs far outweighs any likely impacts from advances in specific treatments.

## “Short Limbed” Dogs and IVDD

Extreme differences in limb length define many of the dog breeds around the world, and the association between specific “short-legged” breeds and premature intervertebral disc degeneration has been documented since the early twentieth century [referenced in (4, 5)] Skeletal dysplasia is a general term describing abnormalities of growth and development of cartilage and/or bone and associated alterations in stature (6, 7). The molecular genetic underpinnings of limb length variability in dog breeds are becoming more completely understood although many unexplained types of skeletal dysplasia remain. Several skeletal dysplasias in specific dog breeds have been associated with mutations in members of the collagen gene family or its binding proteins (8–10), fibrilin related protein (11), as well as an altered sulfate transporter protein (12). However, overexpression of *FGF4* associated with insertion of *FGF4* retrogenes on CFA12 and CFA18 appear to have broader influences on limb length across many breeds and are the only genes to have been implicated in body size in across-breed association studies (3, 13–16). While many breed specific mutations are considered undesirable (8–10, 12) some of these genes have been under positive selection in specific breeds due to their effects on height, despite associated pathologies including glaucoma and IVDD (11, 13).

Terminology applied to skeletal dysplasia subgroups can be confusing; the term chondrodysplasia covers a broad group of skeletal dysplasia disorders in humans with abnormal development of the endochondral components of the skeletal system (present at birth) and has been used to describe extreme differences in limb length in several dog breeds, such as the Basset Hound, Dachshund, and Pekingese (14). Historically, the term chondrodystrophy has been applied as a more general terminology to include terms such as chondrodysplasia. In the veterinary literature it has come to be used as a term describing “short limbed” dogs with skeletal dysplasia that additionally have progressive degeneration of the intervertebral disc after birth, with the progressive nature of the IVDD informing the use of the term “dystrophy” (4, 5, 17). While the genetic alterations listed above have all been associated with altered limb length, only the overexpression of *FGF4* secondary to retrogene insertion on CFA12 has also been specifically associated with premature degeneration of the intervertebral disc (13).

## The *FGF4* Gene

FGF4 is one of a family of 18 secreted canonical FGF proteins that interact with 4 signaling tyrosine kinase FGF receptors (FGFR1-4) (18). The FGF4 subfamily (FGF4,5,6) bind to receptors expressed predominantly in mesenchymal tissues (FGFR1c, 2c, 3c, 4), and as with most FGFs they have important roles in early stages of embryonic development and organogenesis (18). *Fibroblast Growth Factor 4* is most highly expressed in the embryonic ectoderm, axial, paraxial, and lateral plate mesoderm, and tail bud (19). Later in development, *Fgf4* is highly expressed in the apical ectodermal ridge of the developing limb bud, as well as somites, which go on to form the vertebral column and non-nuclear components of the intervertebral disc (19–21). FGF signaling is required for proper embryonic axial growth and segmentation and *Fgf4Fgf8* murine hypomorphs are characterized by altered vertebral morphology and smaller limb buds (20, 22). In a mouse model, creation of a gain of function *Fgf4* copy to replace an inactive *Fgf8* gene was able to rescue limb development; however, it also caused abnormal tissue deposition and postaxial polydactyly, highlighting that levels of FGF proteins throughout embryonic development must be properly controlled for normal limb formation (23). FGF signaling is important in development of the ear, and both *Fgf4* and *Fgfr1* are expressed in embryonic structures that give rise to the pinna (19, 24). Hypomorphic alleles of *Fgf4* and *Fgfr1c* (25, 26) both result in reduced pinna size although it is unclear whether FGF4 overexpression specifically results in increased pinna size as is common in many chondrodystrophic dog breeds. Expression of *Fgf4* is not documented specifically in the nucleus pulposus during development (19, 27, 28), however FGF signaling is involved in the differentiation of notochordal cells (29), is present in the developing end plate and annulus fibrosus (28) and mutual interaction between the notochord and vertebral bodies are instrumental in the proper formation of the IVD (21).

## *FGF4* Retrogenes

Two separate retrogenes, derived from the parental *FGF4* gene on CFA18, have been described in dogs resulting in various degrees of skeletal dysplasia and disproportionate dwarfism (13, 14). An *FGF4* retrogene on CFA18, 25 Mb from the parental gene, associated with marked limb length variation (14), and an *FGF4* retrogene on CFA12 associated with chondrodystrophy characterized by moderate variation in limb length and an odds ratio of 51.23 (95% CI = 46.69, 56.20) for Hansen Type I intervertebral disc disease (13). The CFA12 *FGF4* retrogene was identified concurrently by separate genome wide association studies investigating alteration in limb length in Nova Scotia Duck Tolling Retrievers and with IVDD across breeds (13). The same chromosomal region had previously been identified associated with limb morphology in Portuguese Water Dogs and intervertebral disc calcification in Dachshunds without defining a causative mutation (30, 31). Comparatively, activating mutations of the FGFR3, one of the receptors for FGF4, are responsible for some of the most common causes of disproportionate dwarfism in humans including achondroplasia (6, 32). Intervertebral disc degeneration is also a common finding in human achondroplasia, however histopathological characterization is lacking, and many factors including vertebral malformations, spinal canal stenosis and secondary degenerative changes are a major component of disease pathology (33).

**Figure 1:**
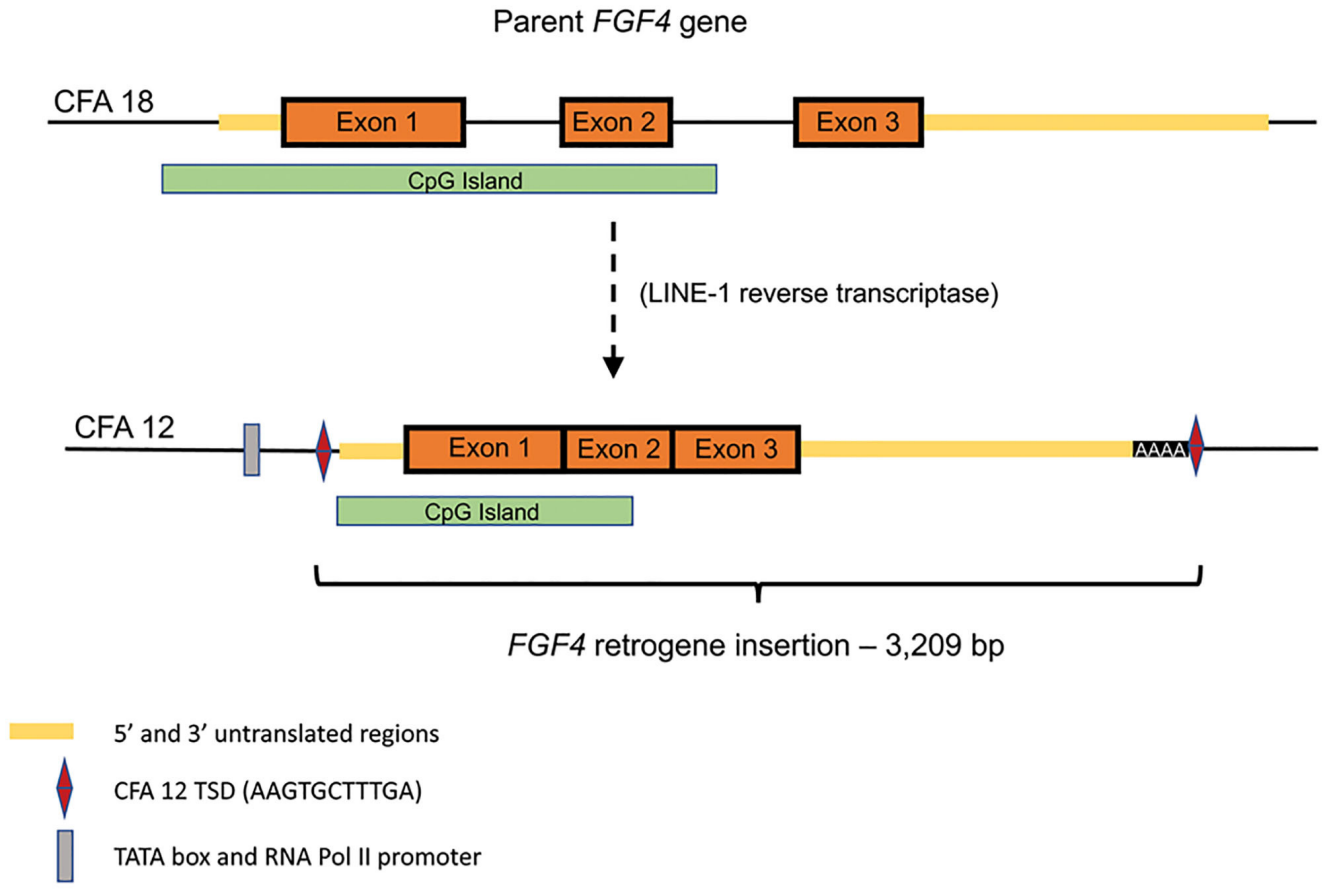
Schematic of endogenous *FGF4* (CFA18) retrotransposition to CFA12:33,710,178 (canFam3): Predicted TATA box at chr12:33,709,940-947 (canFam3) and predicted RNA Pol II promoter at chr12:33,709,964-976 (canFam3) are shown. The retrogene insert includes all of the predicted 3′UTR, followed by 42 adenine residues and the duplicated target site duplication (TSD) sequence (AAG TGC TTT GA). Retrotransposed sequence also includes a large CpG island.

Retroposition is a gene duplication mechanism that utilizes an RNA intermediate to randomly insert intronless retrocopies of genes into the genome following reverse transcription (Figure 1) (34, 35). Reverse transcriptase activity can be provided from a variety of sources, however in mammals it is most commonly associated with long interspersed nuclear elements (LINEs) (35). LINEs are one of several types of transposable DNA sequences that have the ability to change their position within a genome. LINEs utilize their own reverse transcriptase activity to copy and paste themselves into new locations, however this activity may also create DNA copies of mRNA from functional genes which, if inserted, may result in retrogene copies. Both of the canine *FGF4* retrogenes appear to have arisen from RNA retrotransposed by LINE-1 integrase and reverse transcriptase, including flanking target site duplications (TSDs) and polyA tracts (class 1 templated sequence insertion polymorphism) (36).

Retrocopies have historically been considered to be inactive in most cases due to lack of appropriate regulatory elements as well as genetic alterations that remove open reading frames, and documented association with disease states is relatively uncommon. However, retrotransposition is believed to play an important role in genome evolution (35) and as the field develops, examples of disease related retrotransposition are likely to increase in frequency. Currently only the CFA12 and CFA18 *FGF4* retrogenes have been associated with clinical phenotypes in dogs.

The CFA12 *FGF4* retrogene is 3,209 bp long (Gen Bank accession no. MF040221) and consists of the parental chromosome 18 *FGF4* exons (Figure 1) and a majority of the 5′-untranslated region (UTR) including the transcription start site and cis-regulatory elements including a TATA box, RNA Pol II promoter sequences and many conserved transcription factor binding sites. The insertion is at 33.7 Mb between the *OGFRL1* and *RIMS1* genes. [The CFA18 *FGF4* retrogene is a similar size (2,665 bp) with common 5′ UTR and exons but a shorter 3′ UTR and is inserted within a LINE element on the parental chromosome 18]. The CFA12 *FGF4* retrogene is transcriptionally active resulting in a 20-fold increase in FGF expression in neonatal intervertebral disc from dogs homozygous for the CFA12 *FGF4* retrogene insertion compared to disc from neonatal dogs with just the parental *FGF4* genes (13). Although the CFA12 *FGF4* retrogene was inserted near sequences with promoter properties, it is more likely that the *FGF4*-associated CpG island (Figure 1) included in the retrogene and shared with other species, including humans, is driving expression (37). CpG islands are genomic regions with a higher frequency of C-G dinucleotides than the genome average, often co-localizing with gene promoters and having important roles in transcriptional regulation. In fact, retrogene expression has been shown to be dependent on the genomic context of its insertion and contribution of CpG islands more than the use of nearby promoters (38). Seven of eight genes in the direct neighborhood of the CFA12 *FGF4* insertion are actively expressed in neonatal IVD and vertebral body (13) suggesting that the retrogene was inserted in a gene milieu conducive to expression in IVD. Specific embryonic expression of FGF4 in dogs with 2, 4, or 6 copies of the gene (from parental, or either retrogene) is to be defined, however quantitative and/or tissue specific differences associated with the CFA12 *FGF4* retrogene expression may explain the IVD-associated phenotype seen with CFA12 but not the CFA18 *FGF4* retrogene expression.

## IVDD and *FGF4* Retrogenes

Although we have little ancient historical data documenting IVDD prior to the late nineteenth century (39), we do know that descriptions of short-legged dog breeds go back over 4000 years (40). These include depiction of a short-legged dog on a tomb wall in Egypt (2000 BC), ceramic works of the Colima Dog in Mexico from AD 300-600, the description of the Turnspit in the first English dog book in 1576, and the description of the first Dachshund in Germany in 1735. Although the genetic makeup of these dogs is unknown, it is likely that one or both of the *FGF4* retrogenes have been involved in the generation of these ancient short legged phenotypes. Retrotransposition of the *FGF4* gene has occurred at least twice in recent history, although the lack of accumulation of mutations in either retrocopy suggest these events are still relatively recent in genomic evolutionary terms. Once the *FGF4* retrogene(s) appeared and produced an obvious phenotype, strong selection was likely applied to retain them.

**Figure 2:**
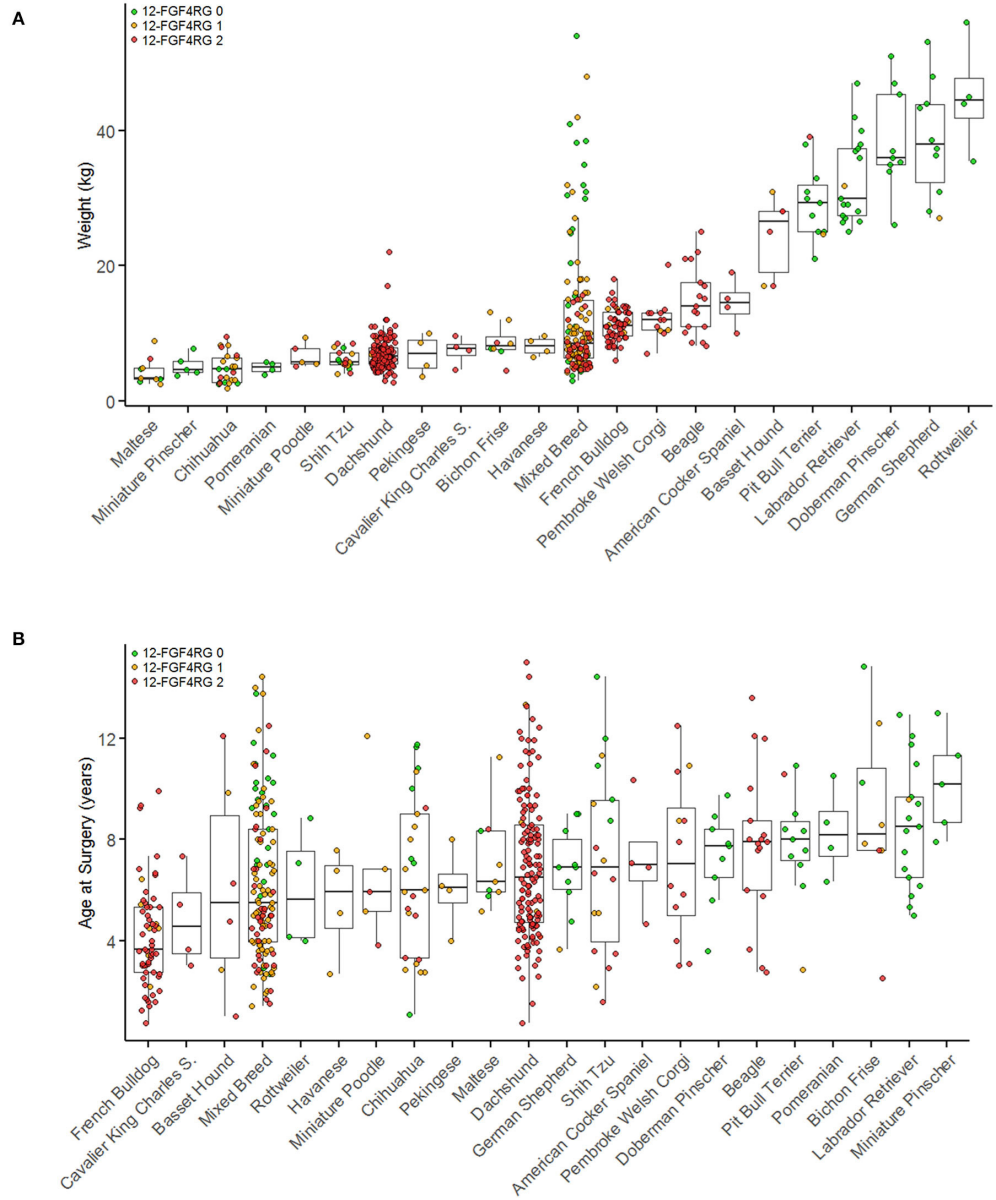
Breed and genotype distribution of surgical IVDD cases by body weight **(A)** and by age at surgery **(B)**. Breeds with fewer than four cases are not included in this figure. Breeds are plotted in order of ascending median weights **(A)** or age **(B)** and colored by CFA12 *FGF4* retrogene genotype. Red indicates two copies of each retrogene, orange indicates one copy, and green indicates zero copies. [Modified from Batcher et al. (42)].

Differential expression of the CFA12 and CFA18 *FGF4* retrogenes generally reflects the morphological phenotype and susceptibility to IVDD we recognize in clinical practice (Table 1, Figure 2). Breeds carrying one *FGF4* retrogene copy (CFA12 or CFA18) tend to have skeletal dysplasia with moderately short limbs, while those carrying both copies, such as Dachshunds, Corgis and Bassett Hounds, have the most severe form of disproportionate dwarfism. The breeds with a higher frequency of the CFA12 *FGF4* insertion are the same breeds identified in the last 50 years as being predisposed to IVDD (4, 5, 41). Although the CFA18 *FGF4* retrogene is found commonly in breeds that also have the CFA12 *FGF4* retrogene and IVDD (Dachshunds, Corgis, Bassett Hounds), it does not appear to be directly associated with development of IVDD since several of the highly susceptible short legged breeds carrying the CFA12 *FGF4* retrogene such as Beagles, French Bulldogs and Cocker Spaniels do not carry the CFA18 *FGF4* retrogene and interestingly contrast with the short legged breeds such as Cairn Terriers and West Highland White Terriers that are rarely reported to have IVDD and carry only the CFA18 *FGF4* retrogene (5, 13, 42).

**Table 1:** *FGF4* retrogene allele frequencies.

		**CFA12** ***FGF4*** **Retrogene**				**CFA18** ***FGF4*** **Retrogene**			
**Breed**	**Total**	**0**	**1**	**2**	**Frequency**	**0**	**1**	**2**	**Frequency**
Beagle	29	0	0	29	1.00	19	0	0	0
Cavalier King Charles Spaniel	25	0	0	25	1.00	6	0	0	0
Clumber Spaniel	6	0	0	6	1.00	6	0	0	0
Dachshund	509	0	30	479	0.97	3	9	482	0.98
Cocker Spaniel, American	13	0	1	12	0.96	6	0	0	0
Cocker Spaniel, English	14	0	1	13	0.96	14	0	0	0
Bulldog, French	113	0	14	99	0.94	71	2	0	0.01
Dandie Dinmont Terrier	28	1	5	22	0.88	0	1	27	0.98
Welsh Corgi, Pembroke	63	3	15	45	0.83	0	2	61	0.98
Welsh Corgi, Cardigan	7	1	1	5	0.79	0	2	4	0.83
Skye Terrier	13	2	2	9	0.77	0	0	9	1.00
Basset Hound	38	3	20	15	0.66	1	4	27	0.92
Pekingese	32	5	15	12	0.61	1	1	22	0.94
Coton de Tulear	14	3	6	5	0.57	0	1	12	0.89
Poodle, Miniature and Toy	119	28	46	45	0.57	38	10	5	0.19
Springer Spaniel, English	23	10	8	5	0.39	13	0	0	0
Nova Scotia Duck Tolling Retriever	172	69	87	16	0.35	7	0	0	0
Shih Tzu	128	69	42	17	0.30	0	3	16	0.92
Bichon Frise	79	51	23	5	0.21	1	2	5	0.75
Mixed Breed	678	477	148	53	0.19	495	118	65	0.18
Chihuahua	224	170	46	8	0.14	4	15	43	0.81
Jack Russel Terrier	14	11	3	0	0.11	3	1	1	0.3
Danish Swedish Farmdog	29	23	6	0	0.10	12	0	0	0
Chesapeake Bay Retriever	41	34	7	0	0.09	10	0	0	0
Brittany	17	17	0	0	0.07	6	0	0	0
Maltese	95	85	8	2	0.06	0	0	27	1.00
Pinscher, Miniature	9	8	1	0	0.06	9	0	0	0
Schnauzer, Miniature	9	8	1	0	0.06	9	0	0	0
Scottish Terrier	12	11	1	0	0.04	0	1	6	0.93
Australian Shepherd	48	45	3	0	0.03	37	0	0	0
Yorkshire Terrier	15	14	1	0	0.03	0	0	5	1.00
German Shepherd Dog	25	24	1	0	0.02	15	0	0	0
Labrador Retriever	38	37	1	0	0.01	28	0	0	0
Australian Cattle Dog	12	12	0	0	0	5	0	0	0
Bernese Mountain Dog	11	11	0	0	0	5	0	0	0
Border Collie	5	5	0	0	0	5	0	0	0
Border Terrier	6	6	0	0	0	6	0	0	0
Boston Terrier	7	6	1	0	0	7	0	0	0
Bull Terrier	5	5	0	0	0	5	0	0	0
Bulldog, English	13	13	0	0	0	5	0	0	0
Cairn Terrier	10	10	0	0	0	0	1	9	0.95
Doberman Pinscher	23	23	0	0	0	10	0	0	0
Fox Terrier	12	12	0	0	0	7	0	1	0.13
Golden Retriever	14	14	0	0	0	4	0	0	0
Great Dane	13	13	0	0	0	5	0	0	0
Irish Setter	8	8	0	0	0	5	0	0	0
Newfoundland	14	14	0	0	0	5	0	0	0
Norwich Terrier	19	19	0	0	0	3	0	16	0.84
Poodle, Standard	55	55	0	0	0	30	0	1	0.03
Pug	9	9	0	0	0	7	0	0	0
Rottweiler	15	15	0	0	0	5	0	0	0
Shetland Sheepdog	13	13	0	0	0	5	0	0	0
Siberian Husky	13	13	0	0	0	5	0	0	0
St. Bernard	12	12	0	0	0	5	0	0	0
Weimaraner	14	14	0	0	0	5	0	0	0
West Highland White Terrier	10	10	0	0	0	0	0	8	1.00
Whippet	6	6	0	0	0	5	0	0	0

## *FGF4* Gene Dosage

**Figure 3:**
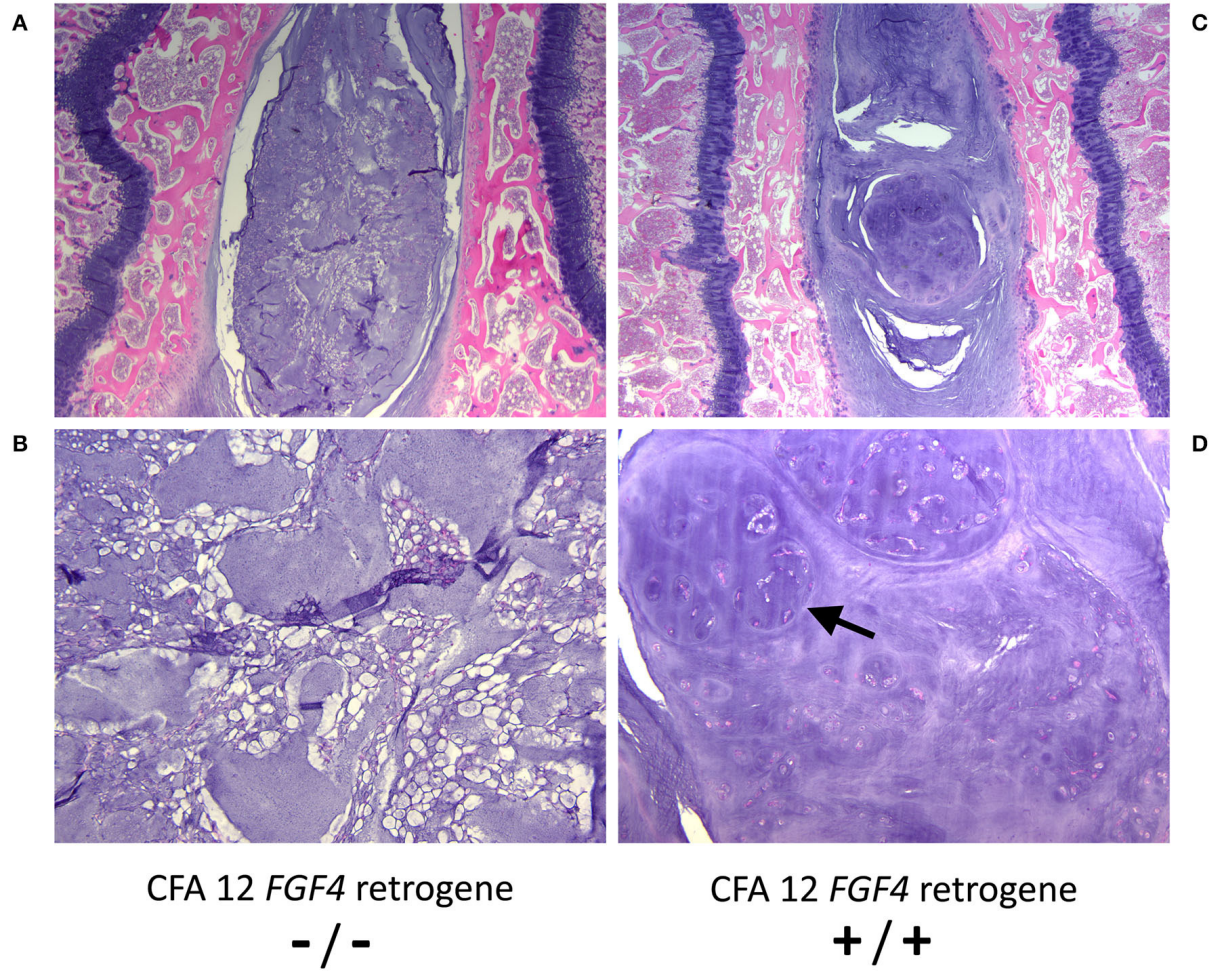
Histopathological images of nucleus pulposus from 10 week-old control (CFA12 *FGF4* retrogene –/–) **(A, B)** and 10 week-old homozygous CFA12 *FGF4* retrogene (CFA12 *FGF4* retrogene +/+) Nova Scotia **(C, D)** Duck Tolling Retriever puppies. The control nucleus pulposus has numerous normal physaliferous notochordal cells with foamy to vacuolated cytoplasm and often stellate cytoplasmic processes. By 10 weeks the CFA12 *FGF4* retrogene-carrying dog's nucleus pulposus **(B)** consists predominantly of round to ovoid chondrocyte-like cells arranged either individually or in nodular clusters (arrow) associated with a dark purple chondroid matrix. Normal notochordal cells are rare. Both dogs have zero copies of the CFA18 *FGF4* retrogene. H&E stain. Magnification **(A, C)** = 20X; **(B, D)** = 100X.

While it is clear that the CFA12 *FGF4* retrogene is highly associated with premature chondroid metaplasia and degeneration of the intervertebral discs (13, 42, 43), assessing the effects of the CFA12 *FGF4* retrogene is complicated as many high susceptibility breeds also carry the CFA18 *FGF4* retrogene, and many breeds susceptible to IVDD are homozygous for both of the *FGF4* retrogenes making assessment of relative risk challenging. It is also probable that there are additional modifying or causative genetic loci as well as morphological and environmental factors contributing to the overall clinical presentation of IVDD across breeds (44–50). We do know that the presence of the CFA12 *FGF4* retrogene alone is sufficient to cause loss of the normal physaliferous notochordal cells and replacement of the nucleus pulposus by cartilaginous material in puppies as young as 10 weeks of age (Figure 3) (43). Sufficiency was demonstrated in Nova Scotia Duck Tolling Retriever dogs homozygous for the CFA12 *FGF4* retrogene with no CFA18 *FGF4* retrogene and compared to breed matched controls with neither retrogene (43). The effect of the CFA12 *FGF4* retrogene on IVD degeneration also appears to be dominant since IVDD is seen in dogs that are homozygous or heterozygous for the CFA12 insertion (13, 42).

### Allele Frequency

Genotyping of over 3,000 dogs from 75 breeds showed that the CFA12 *FGF4* retrogene was present in 40 breeds, the CFA18 *FGF4* retrogene in 32 breeds and both retrogenes in 23 breeds (Table 1) (42). The CFA12 *FGF4* retrogene has an extremely high allele frequency (>90%) in the general population of breeds such as Beagles, Dachshunds, French Bulldogs, and most spaniel breeds, and not surprisingly, this frequency increases when looking at the population of dogs that present with clinical IVDD (42). Batcher et al. investigated 569 dogs presenting for decompressive surgical treatment of intervertebral disc disease and described differences in cases typified by calcified/mineralized disc herniations compared to those with “fibrous” type herniation with respect to retrogene frequency. Consistent with typical clinical neurological caseload, 75% of all cases involved throacolumbar discs, and affected dogs frequently had 1 or 2 copies of the CFA12 *FGF4* retrogene (allele frequency 0.636). Consistent with the skeletal dysplasia phenotype common to both retrogenes, presence of 1 or 2 copies of either the CFA12 or CFA18 *FGF4* retrogenes was also associated with “smaller” dogs (based on body weight).

### Age Related Factors

Defining age of onset of IVDD is challenging for many reasons, not least because histopathological evidence of premature degeneration is already present in affected dogs before 1 year of age (4, 41, 43). Presence of 1 or 2 copies of the CFA12 *FGF4* retrogene is associated with a younger age of presentation for decompressive surgery (mean 6.1 vs. 8.5 years) (Figure 2) (42). Interestingly and as reported previously, French Bulldogs (51, 52) that are essentially fixed for the CFA12 *FGF4* retrogene had a significantly younger age at surgery (median 3.7 years) compared to other breeds homozygous for the CFA12 *FGF4* retrogene such as Dachshunds (median age 6.5 years) (42). Number of CFA 12 *FGF4* retrogene copies does not appear to affect age at presentation for surgery (42). The role of the CFA 18 *FGF4* retrogene is less clear; a single copy was associated with a younger age at surgery, however no difference was seen comparing 2 copies vs. zero copies or 1 and 2 copies (42).

### Calcification: Type I vs. Type Ii

Calcification/mineralization of intervertebral discs, either surgically or radiographically, is typically used as a surrogate for the presence of Hansen type I vs. Hansen Type II disc disease since it is rarely present in the latter (5, 53). Consistent with previous reports (5, 53–55), studies looking at IVD calcification and CFA12 *FGF4* retrogene frequency also described dogs with calcified (Type I) IVD to be significantly smaller (median 8.1 vs. 25 kg) and to have a significantly younger age at presentation for surgery (5.5 vs. 9 years) compared to non-calcified IVD (Type II) dogs (42). Supportive of the proposed role of the CFA12 *FGF4* retrogene in premature chondroid degeneration and associated mineralization of the IVD, CFA12 *FGF4* retrogenes are more common in surgically treated dogs with evidence of calcification (allele frequency 0.77) compared to surgically treated animals with fibrous/non-calcified disc herniation/protrusion (allele frequency 0.149) (42).

### Radiographic Screening

Degree of calcification of the IVD has been shown to be heritable in Dachshunds (56–58) and associated with risk of clinical IVDD in Dachshunds and Pekingese dogs (47, 55, 59, 60). Radiographic screening based on IVD calcification severity scores has been used historically as a potential tool for selective breeding specifically within the Dachshund breed. There are many variables that affect radiographic presence of IVD calcification, and temporal factors can play a major role with appearance and resolution of calcified discs over time (54, 61). Prospective screening with defined time points, that may be breed specific, is important for optimal “scoring” of at-risk dogs. A retrospective analysis of presence or absence of IVD calcification (uncontrolled for age at time of assessment) showed that the observation of calcified discs was significantly more likely in dogs with 2 copies of the CFA12 *FGF4* retrogene (84.8%) compared to 1 copy of the CFA12 *FGF4* retrogene (63.8%) compared to zero copies (18.5%) (42). Multivariable logistic regression identified presence of the CFA12 *FGF4* retrogene as the main contributor to disc calcification with 2 copies of the CFA12 *FGF4* retrogene increasing the odds of disc calcification by a factor of 2.5 compared to 1 copy (42). Presence or absence of the CFA18 *FGF4* retrogene had no significant effect on odds of observing IVD calcification (42).

Decrease in incidence of calcified discs following selective breeding of Dachshunds based on radiographically defined calcification scores has unfortunately been limited (57). Many variables may be influencing progress including precision of scoring (62), limited application and compliance within the breeding population as well as limitations in correlating visual radiographic criteria to underlying genetic status (55, 63). The original mapping of IVD-associated calcification to the CFA12 *FGF4* retrogene location on chromosome 12 was done using high vs. low radiographic calcification scores in Dachshunds (31). Given the extremely high CFA12 *FGF4* retrogene allele frequency in Dachshunds, the low calcification phenotype most likely defined a population of heterozygous (possibly wild type) Dachshunds. This population would be necessary for a successful genome mapping study and is consistent with the CFA12 *FGF4* retrogene dosage effects on radiographically apparent calcification subsequently demonstrated following identification of the CFA12 retrogene (42). Pilot data from a small population of Danish Wire Haired Dachshunds that appear to segregate the CFA12 *FGF4* retrogene showed an OR of 6.1 for high calcification screening scores associated with either 1 or 2 copies of the CFA12 *FGF4* retrogene (64).

The overall dominant role of the CFA12 *FGF4* retrogene with heterozygous and homozygous animals potentially having overlapping degrees of IVDD and calcification together with the variables above are likely reflected in the difficulty obtaining rapid reduction in disease using radiographic screening. Although additional genetic variables still remain to be defined, screening strategies based on the CFA12 *FGF4* retrogene rather than down-stream phenotypes may offer more tractable selection data for breeding.

### IVDD Relative Risk

Prospective data determining the risk for IVDD associated with presence of the CFA12 *FGF4* retrogene are still to be collected. Assessment in chondrodystrophic breeds such as Dachshunds, Beagles, and French Bulldogs, with very high allele frequencies preludes analysis in retrospective data. However, evaluation of a small group of surgically treated, mixed breed dogs in which segregation of the retrogene occurred and for which historical control data for aged, clinically unaffected dogs was available has been performed (42). Looking at number of copies of CFA12 and CFA18 *FGF4* retrogenes, body weight and sex, only presence of the CFA12 *FGF4* retrogene was found to be significantly associated with presentation for decompressive surgery for IVDD, with no difference between 1 and 2 copies. Based on these findings and looking at mixed breeds and other breeds that also segregated the retrogene (allele frequency <0.5 and >0.05), relative risk for IVDD (presenting for surgical treatment) associated with the CFA12 *FGF4* retrogene ranged from 5.5 in Chihuahuas to 15.1 in mixed breed dogs.

## What we Don't Know

Several aspects of the clinical presentation of IVDD in dogs are not easily explained by a simple presence or absence of the CFA12 *FGF4* retrogene. Clarification of these pathophysiological and clinical variables in the presentation of IVDD is important if genotyping is to be used as a tool for reduction in disease incidence. A more comprehensive picture will also increase breeder and owner confidence for eradication strategies that may potentially require major alterations in breed standards for those breeds with high allele frequencies.

### CFA12 FGF4 Retrogene Dosage

Risk for clinical disease based on heterozygous or homozygous retrogene status may be powerful data to inform selective breeding strategies. Circumstantial evidence would suggest that there could be an effect of dosage since calcification scores have been shown to be related to risk for IVDD in Dachshunds and Pekingese dogs (47, 55, 59, 60) and disc calcification was shown to be correlated with CFA12 *FGF4* retrogene allele frequency (42). The latter study did not however directly demonstrate a gene dosage effect on age at presentation for surgery or relative risk for surgery (in mixed breed dogs) however scoring was a simple presence or absence and important factors such as age and time of imaging (54, 61) were not controlled. Prospective studies looking at genotyped dogs within selected breeds as well as selective breeding trials with heterozygous animals from high allele frequency breeds should provide deeper insight into gene dosage effects.

### Breed and Environmental-Related Variables

Variables within and across breeds are strongly suggestive for additional genetic, environmental, morphological or metabolic influences on the pathophysiology of IVDD in dogs. The presence of the CFA12 *FGF4* retrogene was associated with a variable risk of presenting for surgery for IVDD from 5.5- to 15.1-fold in different segregating and mixed breeds (42) and even within a relatively non-segregating breed such as the Dachshund, differences in disease prevalence have been noted between different breed types (47, 50). Dachshunds are the most extensively studied breed relating to risk factors for IVDD, however looking retrospectively with current genetic data, historical analyses may have been confounded by variable CFA12 *FGF4* retrogene allele frequencies in different populations of animals. These differences reflect preferences for standard or miniature and variable hair characteristics in different countries. Risk factors including neuter status (45, 50), physical conformation (44, 47–50), axial muscle fascicle length (65), ambient temperature (66), hair characteristics (47, 50), and exercise (46, 50) have been reported with variable consistency in findings across studies.

A shorter T1-S1 vertebral column length and shorter limb (calcaneus-patellar) length were associated with increased risk in one study (48) while a second (49) found increased risk with higher body length to height ratios. Although not documented, the CFA12 *FGF4* retrogene would more likely result in shorter vertebrae, and the later study could have been looking at the dominant effect of the CFA12 *FGF4* retrogene on leg length (rather than vertebral length) as the major determinant of ratio changes. However, several genetic factors are likely to be contributing to conformation including CFA12 and 18 *FGF4* retrogenes. Clinically affected Dachshunds were reported to have longer epaxial muscle fascicle lengths in one study, which could be a secondary effect of clinical IVDD; however, it is interesting to note that FGF4 is also a key signaling molecule in the development of myofibers and tendon of epaxial muscles arising from somitic mesoderm (67, 68).

Data on prevalence of IVDD by Dachshund type varies between studies (47, 50), however in the largest study of over 2,000 Dachshunds, decreased prevalence of IVDD was seen in Standard Wire Haired Dachshunds compared to other standard and miniature types (50). This may be a reflection of the heterozygous CFA12 *FGF4* retrogene status of dogs within the Standard Wire Haired population which can be inferred from the successful GWAS mapping using this Dachshund subtype (31) and further supported by pilot genotyping data from Denmark showing ~30% (27/91) of Wire Haired (but not Smooth or Short Haired) subtypes were heterozygous for the CFA12 *FGF4* retrogene (64). Long haired Dachshund subtypes (standard and miniature) had the next lowest prevalence of IVDD and the long haired phenotype is known to be caused by mutation within the *FGF5* gene (likely loss of function based on recessive inheritance) (69, 70). Although *FGF5* gene expression has typically been associated with hair follicle development, it is phylogenetically closely related to *FGF4* and similar to *FGF4* has been shown to be expressed in developing limbs (18, 71). Redundancy across the FGF signaling pathways is common (18) and it is interesting to speculate whether there may be a biological relevance in IVDD with over and under expression of these 2 closely related FGF genes. The genetic mutation resulting in the wire haired phenotype is also potentially relevant to IVDD as this involves an activating mutation affecting expression of the *RSPO2* gene (69). RSPO2 synergizes with the WNT-β catenin pathway (72), an important contributor to IVD development (73) and down-regulation of WNT signaling has been shown to be present in early IVD degeneration (74).

### Breed Related Calcification

Significant variability in presence of radiographically identified disc calcification (accounting for CFA12 *FGF4* retrogene presence and other factors) based on breed has been reported (42). In dogs presenting for IVDD related surgery, 90.5% Dachshunds, 70.6% French Bulldogs, 60.2% mixed breed dogs, and 40.8% “other pure breed” dogs had at least 1 radiographically defined calcified disc at the time of surgery. The Dachshund data are similar to previous reports (55) and even accounting for potential variability associated with age at assessment, there is a clear breed-associated difference unexplained by the CFA12 *FGF4* retrogene alone.

### Breed Related Age Differences

While age at time of surgery for IVDD is significantly lower for CFA12 *FGF4* retrogene dogs as a group (42), differences within the chondrodsytrophic breeds also suggest additional factors in IVDD pathogenesis. Dachshunds and potentially other breeds with high allele frequencies of CFA12 *FGF4* retrogenes presented at an older age for surgery compared to mixed breeds (42). This may reflect additional selection for modifying factors in these high allele frequency breeds resulting in removal of younger-onset dogs from the breeding pool. One can speculate that presence of within-breed “protective/modifying effects could also explain the higher relative risk associated with the CFA12 *FGF4* retrogene in mixed breed dogs. At the opposite end of the spectrum, French Bulldogs have a significantly lower age of disease presentation (42, 51, 52). Unlike potential activation of the WNT pathway by the *ROSP2* gene in Wire Haired dogs, French Bulldogs have down regulation of WNT signaling due to a frameshift mutation in the Disheveled 2 (*DVL2*) gene associated with screw tail and brachycephaly (75). Whether these chondrodystrophic breed associated WNT pathway alterations (in the context of FGF4 overexpression) are clinically relevant to reported IVD-associated down-regulation of WNT signaling (74) remains to be determined, however WNT and FGF signaling pathways cross-talk during a variety of cellular processes (76).

### Modifying Effects of Other Retrogenes

Co-expression of the CFA12 and CFA18 *FGF4* retrogenes is common in many chondrodystrophic breeds making exclusion of effects of the CFA18 *FGF4* retrogene from retrospective data challenging. However, effects of the CFA18 *FGF4* retrogene appear to be modest (42), potentially affecting age at presentation but not relative risk or prevalence of radiographic disc calcification based on multivariable analyses (42). The CFA12 *FGF4* retrogene alone appears to be sufficient to cause premature degeneration of the IVD (43) although histopathological studies of IVD from young dogs of breeds homozygous for the CFA18 *FGF4* retrogene such as Cairn Terriers, West Highland White Terriers (typified by low clinical incidence of IVDD) would provide further insight into the relative effects of the two retrogenes.

The location of a large CpG island in the two *FGF4* retrogenes documented to date may facilitate its expression in other chromosomal locations as well. This provokes the question of how many other times the *FGF4* gene has been retrotransposed and then eliminated by selective breeding, or if additional retrocopies remain in some breeds and are responsible for other morphological phenotypes or contribute to IVDD.

### IVDD in Dogs Without the CFA12 *FGF4* Retrogene

Previous studies have described Hansen type I IVDD in non-chondrodystrophic breeds (77, 78), and zero copies of the CFA12 *FGF4* retrogene were found in 12% (46/378) of dogs presenting for IVDD related surgery and with documented presence of calcified intervertebral discs (42). Breeds represented included Labrador Retriever, Doberman Pinscher, German Shepherd, Pit Bull Terrier, Rottweiler and Pomeranian and the age at time of surgery was 1.5–2 years older compared to dogs carrying the CFA12 *FGF4* retrogene. Type II degeneration of the IVD is typically seen in older non-chondrodystrophic breeds and rarely is associated with IVD calcification (5, 53), however histopathologically defined disc degeneration in chondrodystrophic and non-chondrodystrophic dogs has many similarities (79). It is possible that some older non-chondrodystrophic dogs could present with IVDD disease similar to chondrodystrophic dogs reflecting the heterogeneity seen within both groups, however the population of CFA12 *FGF4* retrogene negative dogs with calcified IVD could also reflect alternative genetic causes of IVDD characterized by calcification but with a later age of onset.

### Human Genetic Correlates

Canine IVDD has been proposed as a potential model for human degenerative disc disease over many years (53, 80). Specific human disease conditions such as achondroplasia, where underlying genetic causes (*FGFR3* gain-of-function mutations) are defined, may have specific molecular mechanistic similarities to *FGF4* overexpression and IVDD (potentially signaling through FGFR3) in dogs. However, less definitive data are available regarding genetics of IVDD and associated low back pain and sciatica in the general human population. Heritability of risk factors for IVDD in humans was initially established using twin studies (81–84) and a variety of candidate gene polymorphisms have been associated with several aspects of IVDD in a variety of ethnic and age-related groups [reviewed in (85)]. Genome-wide association studies similar to those defining the *FGF4* retrogenes in dogs have yielded several potential variants associated with lumbar disc degeneration, sciatica, or “back pain,” mostly involving intronic, regulatory or intergenic polymorphisms with no defined causative genetic loci to date (85). It is yet to be determined whether these non-coding polymorphisms may be markers for retrotransposition events similar to findings in dogs with IVDD.

The most critical unanswered question relating to the CFA12 *FGF4* retrogene, is what will be the impact of its discovery on the incidence of IVDD in 10–20 years. While it is clear that there are likely to be additional modifying factors, both genetic and environmental, the evidence that the CFA12 *FGF4* retrogene is a major factor in the development of IVDD in chondrodystrophic dog breeds is compelling. As causative variants for diseases associated with breed morphological traits are defined, the veterinary profession is being forced to face ethical decisions where the primary mission of the profession (“.prevention and relief of animal suffering…”) may sometimes conflict with dog breeding phenotypic goals.

Positively, some degree of segregation of the CFA12 *FGF4* retrogene has been seen in almost all IVDD affected breeds, even those with high allele frequencies. In breeds with a high degree of segregation it should be possible to reduce or eliminate the retrogene from the population.

Even in high allele frequency breeds, such as the Dachshund, there are frequency differences between populations [0.98 in USA/UK samples and 0.94 in Swiss samples (42)], indicating that some populations may be less homozygous than others, and segregation may be much higher in specific types such as Wire Haired Dachshunds. Many morphological traits are polygenic in nature (2, 3) and selection for a short-limbed phenotype has likely driven selection of dogs harboring *FGF4* retrogenes. In high IVDD risk breeds such as Dachshunds, Bassets, Corgis and Pekingese that have both the CFA12 and CFA18 *FGF4* retrogene, breeding away from the CFA12 FGF4 retrogene, while still maintaining the aesthetically desirable shortness in stature contributed by the CFA18 *FGF4* retrogene is possible.

The dominant nature of the CFA12 FGF4 retrogene on IVDD and the very high allele frequency in some breeds means that eradication may be challenging. The Wire Haired Dachshund was developed through crossbreeding with Schnauzer and Terrier breeds which may explain the lower frequency of the CFA12 susceptibility locus and lower incidence of IVDD in some wire haired populations. Long term strategies may require a combination of testing and selection of heterozygous dogs, outbreeding, cross breeding, and alteration in breed standards, maybe with inclusion of additional CFA12 FGF4 retrogene-negative phenotypes within the breed standards. Whatever the future holds, from a veterinary perspective, current data suggest that breeding priorities should be for dogs with fewer copies of the CFA12 *FGF4* retrogene, so that the allele frequency can be reduced.

## Author Contributions

All authors listed have made a substantial, direct and intellectual contribution to the work, and approved it for publication.

## Conflict of Interest

The authors declare that the research was conducted in the absence of any commercial or financial relationships that could be construed as a potential conflict of interest.
